# Case report: Distraction osteogenesis of focal fibrocartilaginous dysplasia and literature review

**DOI:** 10.1016/j.ijscr.2024.109271

**Published:** 2024-01-13

**Authors:** Mehmet Coskun, Deniz Akbulut, Javad Mirzazada

**Affiliations:** a Medistanbul Hospital, Istanbul, Türkiye; b Tatvan Can Hospial, Bitlis, Türkiye; c Medical Park Kocaeli Hospital, Kocaeli, Türkiye

**Keywords:** Focal fibrocartilaginous dysplasia, Ulna, Case report

## Abstract

**Introduction and importance:**

Focal fibrocartilaginous dysplasia (FFCD) is a rare disease of the ulna that requires early surgical intervention.

**Case presentation:**

We present a juvenile case and the outcomes of a corrected deformity. The fibrotic band that adhered to the ulna was resected, the ulna was osteotomized, and then an external fixator was placed for lengthening. The ulna's distal physis line was extended by 18 mm so that it would be the same length as the distal physis line of the radius. Full functional recovery occurred within three months.

**Clinical discussion:**

There have been 22 cases of ulnar FFCD reported in the literature. Our patient is the oldest reported thus far who developed radial head subluxation, but no dislocation occurred.

**Conclusion:**

FFCD is a broad-spectrum disease. Although its course is generally poor for patients with a late diagnosis, it is possible to obtain good results with correction procedures.

## Highlights

• Although FFCD is mostly seen in the proximal tibia, the number of cases in the ulna in the literature is less than 25.
• The excessive tissue excision and bone lengthening over ex-fix were obtained in three months with complete functions.
• The ulnar-type FFCD, which can lead to radial head luxation, was first described by Kazuki in 2005.
• Our patient is the oldest diagnosed patient in the literature with radial head subluxation yet no dislocation.

## 1 Introduction

Focal fibrocartilaginous dysplasia (FFCD) is a rare disease in children and leads to deformity. Although it is mostly seen in the proximal tibia, a small number of cases in the ulna have been reported (fewer than 25 cases). FFCD deformity in the ulna is a progressive disease that leads to shortness of the ulna and radial head dislocation. Surgical correction is recommended at an early age. We present a case of FFCD in a 101-month-old boy.

## 2 Case report

The patient was a 101-month-old boy whose family came to our clinic with complaints of deformity and restriction of movement in his right forearm. The deformity had developed in the last 5 months, and he had no other deformities. The patient was followed up at the pediatric endocrinology clinic in our hospital due to short stature.

The patient's weight was 20 kg (1.44 percentile), and his height was 119 cm (2.68 percentile). His body mass index was 14.12 (7.11 percentile), and his weight-for-height ratio was in the 91.32 percentile. Based on this and comprehensive blood tests, he was diagnosed with structural short stature.

**Fig. 1:**
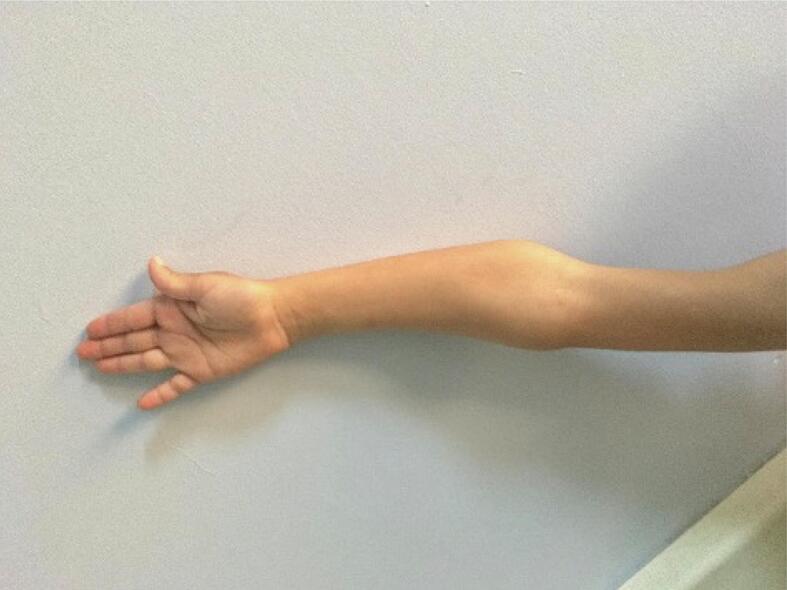
Preoperative right elbow and forearm deformity.

**Fig. 2:**
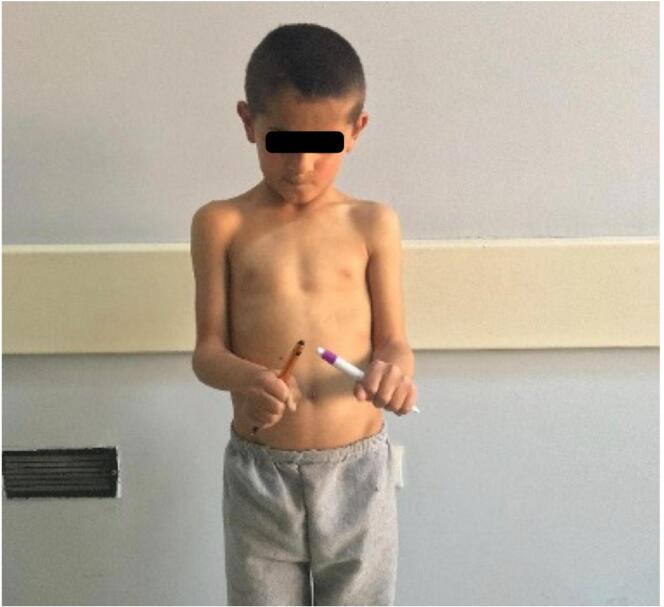
Preoperative limited rotation of the right forearm.

**Fig. 3:**
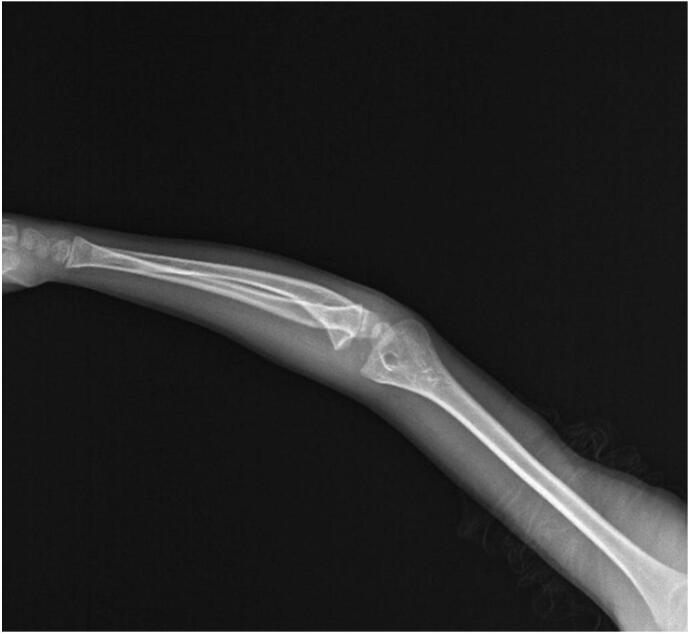
Preoperative deformity of the elbow and forearrm due to the shortness of the ulna.

A prominent varus deformity was observed in the right forearm upon physical examination (Fig. 1). He had normal elbow and wrist flexion and joint-extension ranges of motion (ROMs). His forearm supination was as expected, but his pronation was found to be restricted by 25 degrees (Fig. 2). Plain radiographs showed shortness of the ulna, varus deformity in both the ulna and radius, radial head subluxation, and cortical irregularity in the distal 1/3 of the ulna (Fig. 3). This work has been reported in line with the SCARE criteria [1].

### 2.1 Surgical method

**Fig. 4:**
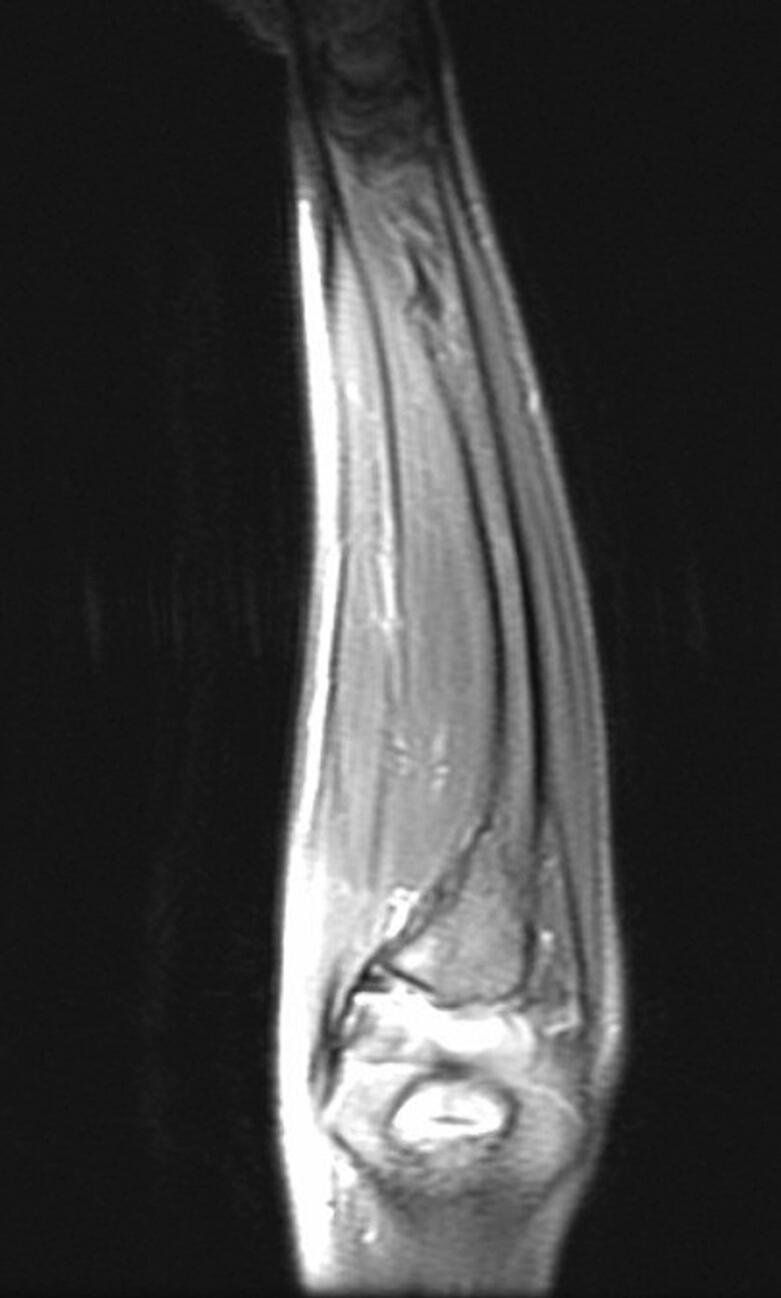
Preoperative MRI indicates the FCD at the distal ulna (sagittal view).

**Fig. 5:**
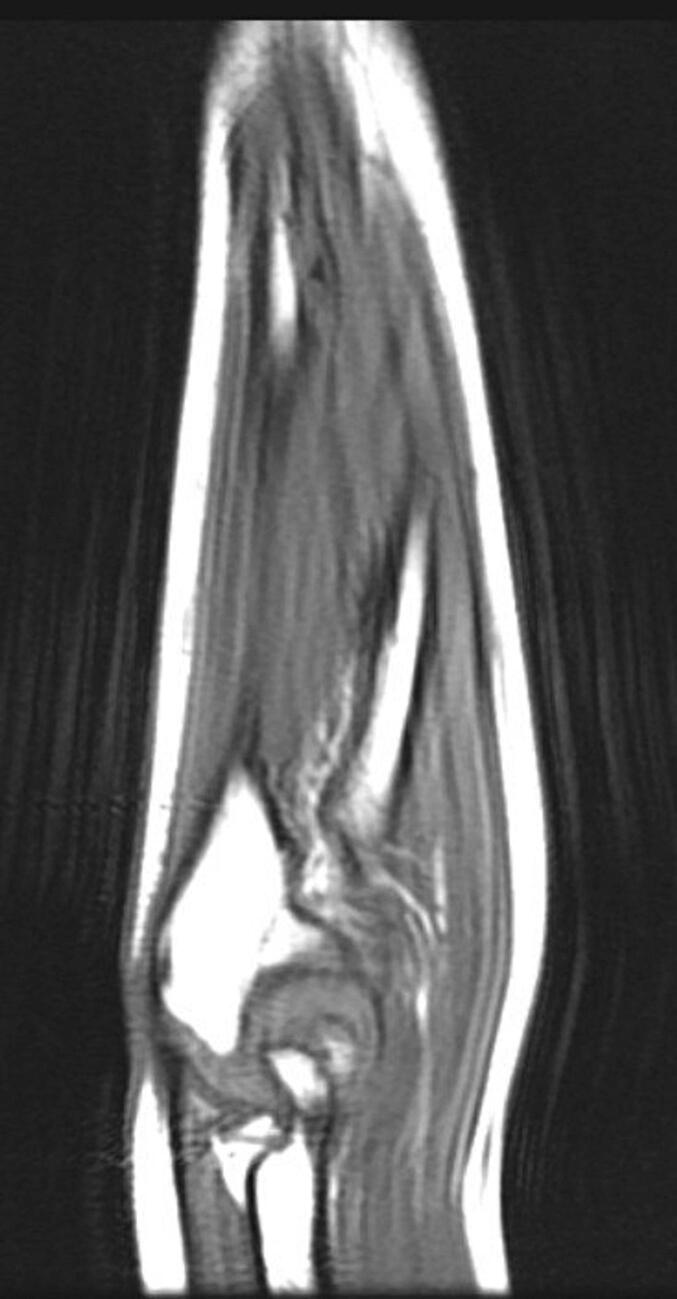
Preoperative MRI indicates the FCD at the distal ulna (frontal view).

**Fig. 6:**
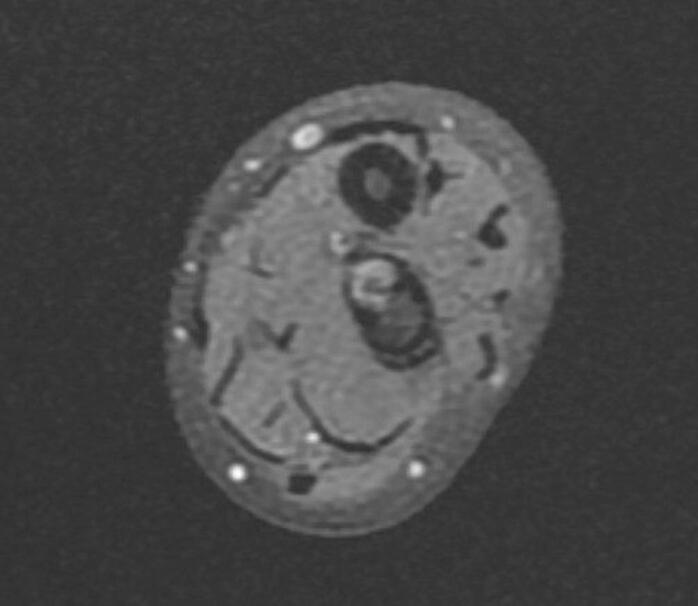
Preoperative MRI indicates the FCD at the distal ulna (axial view).

**Fig. 7:**
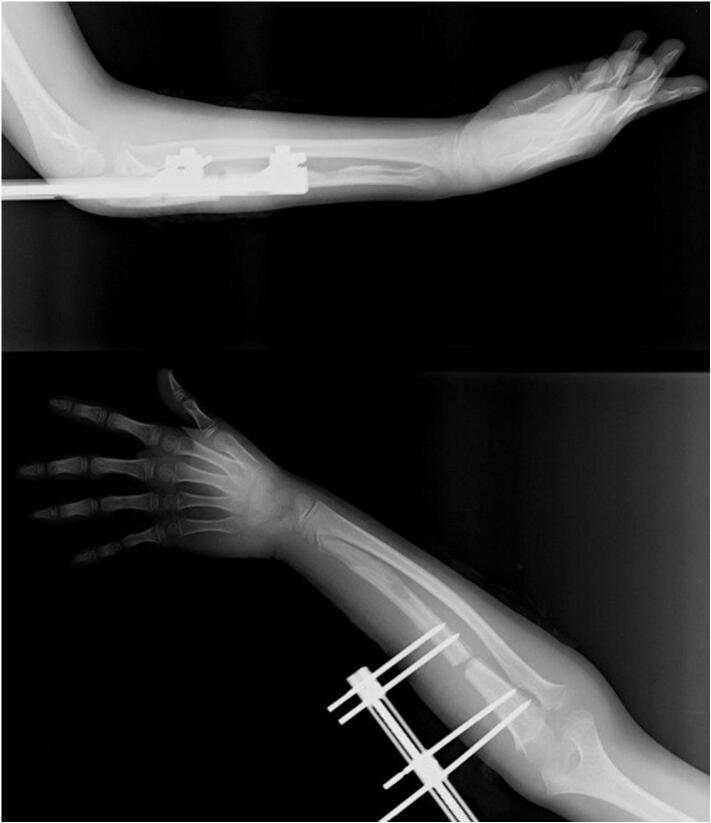
Distraction osteogenesis of the ulnar shaft following the removal of the FCD at distal ulna.

Following a skin incision over the distal 1/3 of the ulna, the ulnar artery and nerve were dissected, and the volar face of the ulna was reached. The fibrotic band-shaped tissue that was observed to adhere to the periosteum distal part of the ulna was removed (Fig. 4, Fig. 5, Fig. 6). Afterward, an osteotomy was performed from the ulna proximal metaphysis, and a rail external fixator was installed to correct the deformity (Fig. 7).

An extension of 1 mm per day was started on the fifth day postoperatively. The ulnar distal physis line was extended by 18 mm so that it would be the same length as the distal physis line of the radius. In post-extension monitoring, the radial head subluxation regressed, and there was no need for osteotomy of the radius.

**Fig. 8:**
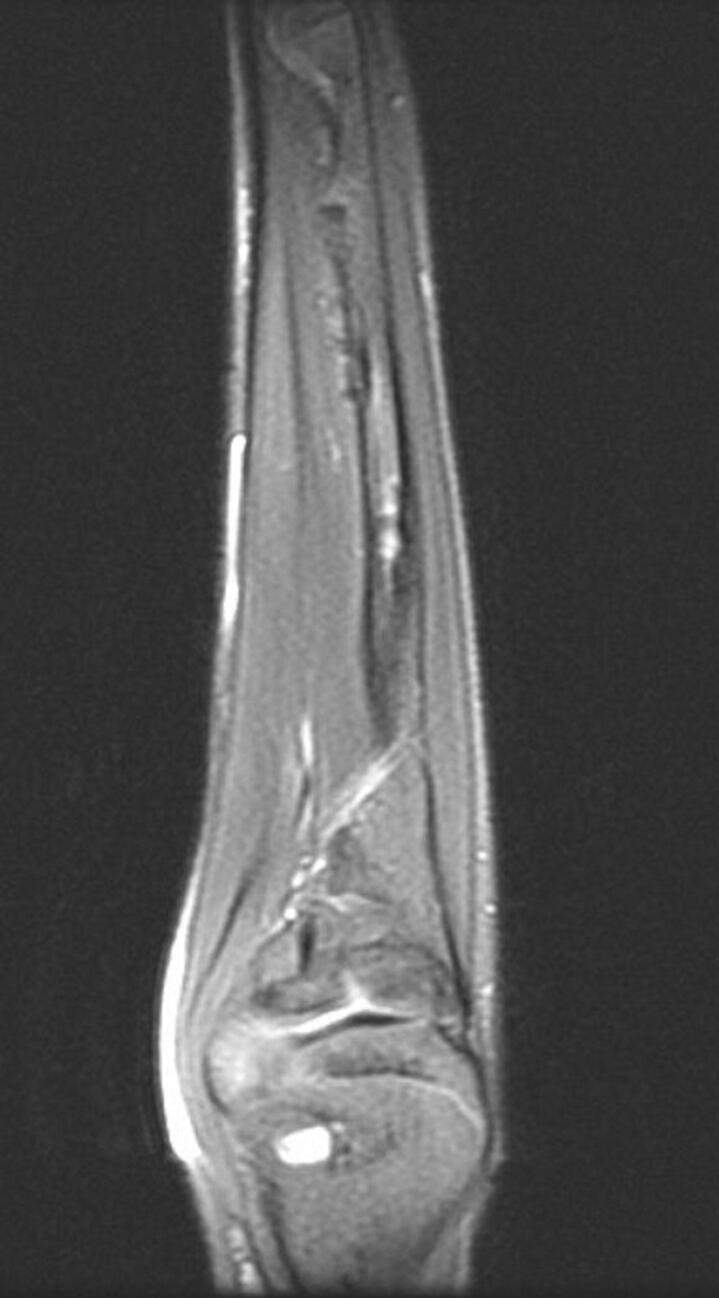
Postoperative MRI indicates the total removal of the FCD (sagittal view).

**Fig. 9:**
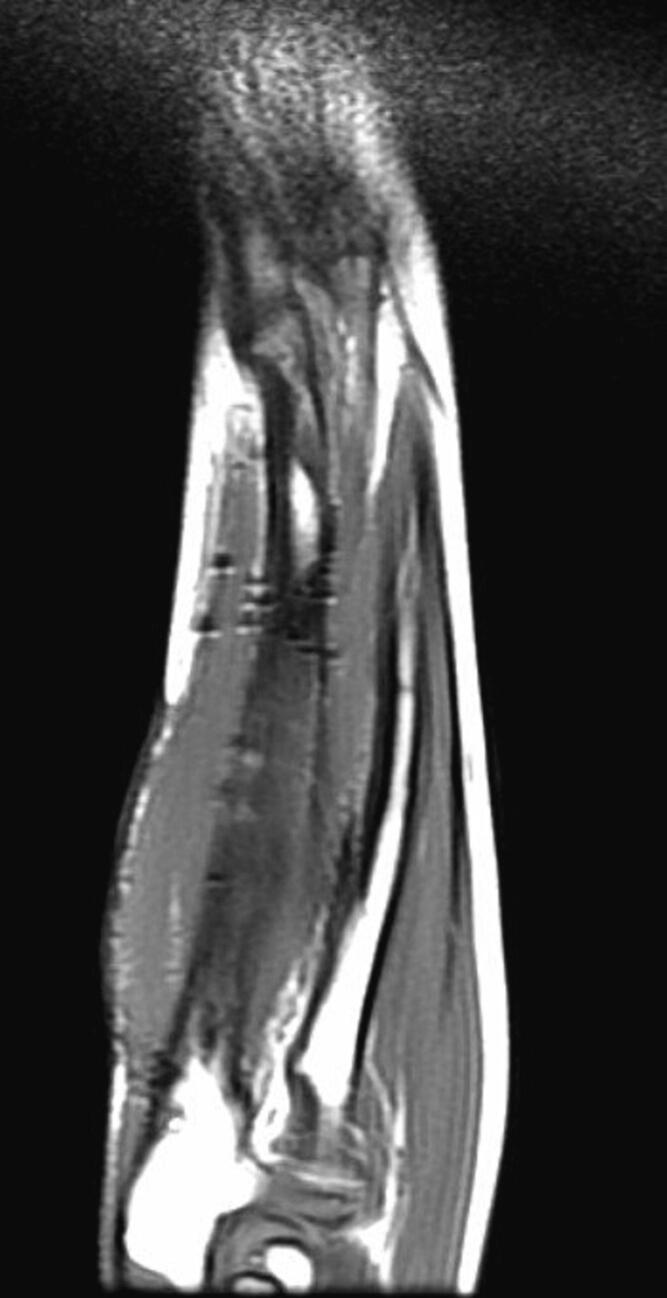
Postoperative MRI indicates the total removal of the FCD (frontal view).

**Fig. 10:**
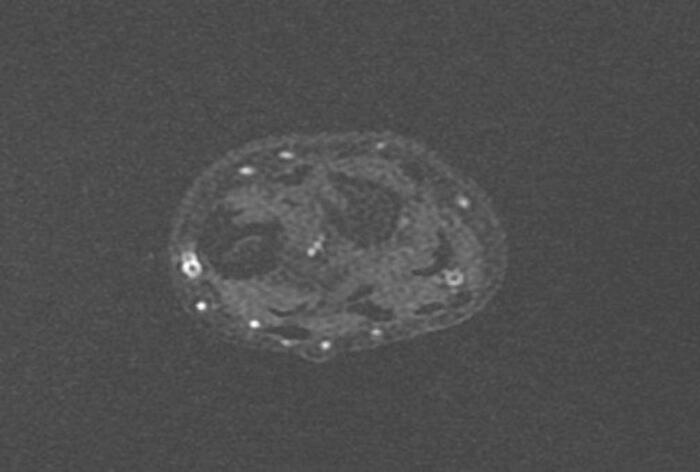
Postoperative MRI indicates the total removal of the FCD (axial view).

**Fig. 11:**
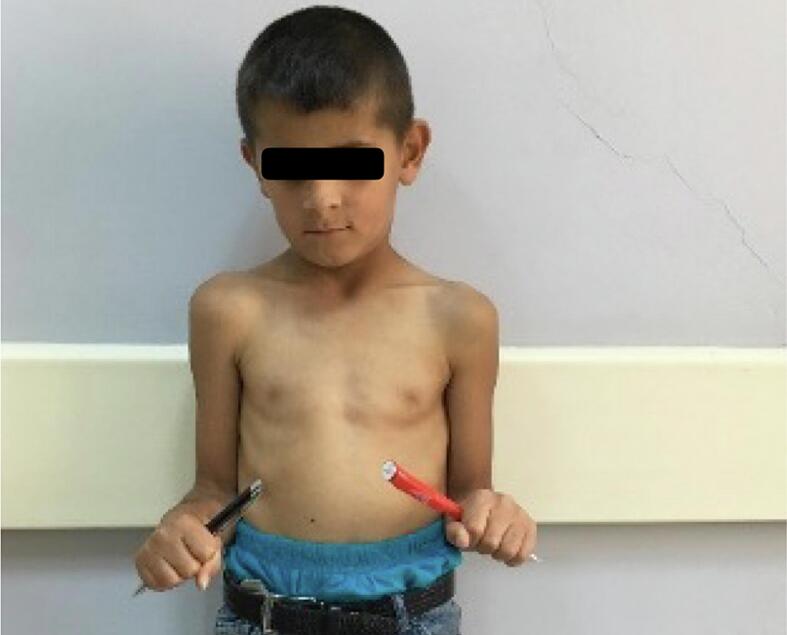
Postoperative full range of rotational motion of the forearm is achieved.

**Fig. 12:**
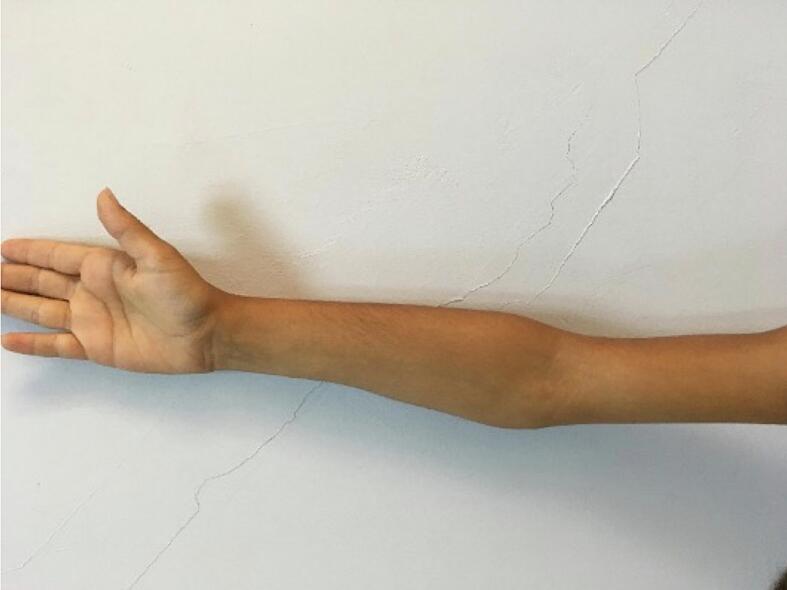
Postoperative adequate correction of the elbow and forearm achieved.

In post-extension physical examination, the 25 degrees of restriction in forearm pronation had regressed, and the forearm ROM was found to be as normal as the opposite limb. After 3 months of extension follow-up, full union was observed, and the rail external fixator was removed. Improvement in the cortical signal was observed in control MRI in the first year postoperatively (Fig. 8, Fig. 9, Fig. 10). As of the 3-year follow-up, recurrent deficit has not been observed (Fig. 11, Fig. 12).

## 3 Clinical discussion

FFCD is a rare disease in children that leads to deformity. Since Bell et al. [2] described the first reported case in 1985 in the proximal tibia, there have been about 100 cases in the literature. It is most commonly observed in the proximal medial tibia. FFCD in the upper extremities is rare, and the number of cases in the literature is fewer than 25 [3,4]. Ulnar-type FFCD can lead to radial head luxation and was first described by Kazuki in 2005 [5].

The etiology of FFCD needs to be clarified. Its pathology can involve tissues such as chondral, ligament, and tendon tissues. The mechanism deformity development involves fibrocartilaginous tissue adhering to the metaphyseal region of the bone, which limits growth with an epiphysiodesis-like effect. This deformity of the ulna leads to secondary deformity of the radius, which leads to increased radius bowing and radial head luxation.

Clinical findings vary according to the affected bone. It is detected within 24–48 months after walking in cases involving the lower extremities and later in cases involving the upper extremities. The most common complaint at clinical presentation is progressive deformity. The affected bone is also adequate in the natural course of the disease. Spontaneous regression is more common in the lower extremities. Jouve et al. [6] state that if spontaneous regression develops, it will occur at an early age. In cases detected in patients under 2 years of age, 6 t months of follow-up can be performed to determine the behavior of the lesion.

Radiographs are pathognomonic for diagnosis. In the ulnar variation of FFCD, a sclerotic lesion around the bone that affects the distal 1/3 of the ulna is observed with a well-circumscribed and oblique elongated lesion in the middle. The ulna is shorter than in the contralateral extremity, and varus deformity is present. Radius deformities secondary to ulna deformities due to shortness and varus deformity are seen in the early stages of the disease, and isolated bowing increases in the radius. In contrast, radio-capitellar joint subluxation and dislocation are seen in later periods.

In MRI examination, a fibro-ligamentous tether can be seen. Ligamentous tethering may be useful for early diagnosis before the development of deformity. The differential diagnosis should exclude ulnar deficiency, tumors involving the ulna distal (fibrous dysplasia), hereditary multiple osteochondromatosis, and infection sequelae [3,[6], [7], [8], [9], [10]].

As stated by Jouve et al. [6], classic findings do not necessitate further imaging or biopsy, so we did not do so for further diagnostics. Treatments for 22 reported cases involved simple observation (6 cases), biopsy (1 case), one-bone forearm reconstruction (5 cases), excision of the fibrous tissue (2 cases), and corrective osteotomies with or without ulnar lengthening (7 cases). Radial head luxation developed in the majority of the patients who were observed, but surgical treatment was not performed due to a lack of functional complaints. With isolated fibrous tissue excision or biopsy, separation of tissue from the adhesion in the bone can be applied at a very early age, which prevents the development of deformity with the effect of epiphysiodes.

One-bone forearm operation is a salvage procedure that is applied in late cases to correct the forearm position and maintain functionality. In addition to distal fibrous tissue excision, ulnar corrective osteotomy is an ideal surgical technique to maintain functionality in cases with or without lengthening deformity. In the treatment of FFCD ulnar variation, surgery is prominent at early ages because the deformity that develops in the ulna causes secondary deformity in the radius and radial head subluxation.

According to Smith et al. [8], the easiest method is to correct the ulna deformity and the congruent radio-capitellar joint. Complications increase with radius-head dislocation in progressive deformities. Radial head dislocation is expected in late presentations of the disease, which was mentioned by Gershkovich et al. [10] and was reported in other cases in the literature. This salvage procedure is usually the only option.

Our patient is the oldest diagnosed patient to be reported thus far in whom radial head subluxation developed but no dislocation has occurred. Due to the limited forearm pronation, ulna correction and extension were required. Complete functional and cosmetic improvement was achieved without the need for a salvage procedure.

## 4 Conclusion

FFCD is a broad-spectrum disease. Ulnar involvement is rare, but it is the most frequently reported affected area in the upper extremities. Unlike other presentations, good follow-up of the radio-capitellar joint and early surgery are recommended due to deformities. Although the course is generally poor for patients with a late diagnosis, it is possible to obtain good results with correction procedures.

## Consent

Written informed consent was obtained from the patient's parents/legal guardian for publication and any accompanying images. A copy of the written consent is available for review by the Editor-in-Chief of this journal on request.

## Ethical approval

Case report does not require ethical approval in our institution.

## Funding

None.

## Author contribution

All the effort was accomplished by the given authors equally.

## Guarantor

The first given author, Mehmet Coskun, is otherwise the mere guarantor in this study.

## Declaration of competing interest

All the authors declare that they have no conflicts of interest. Furthermore, no generative AI technology has been used in this study.
